# A Q-TWiST analysis comparing panitumumab plus best supportive care (BSC) with BSC alone in patients with wild-type *KRAS* metastatic colorectal cancer

**DOI:** 10.1038/bjc.2011.179

**Published:** 2011-05-24

**Authors:** J Wang, Z Zhao, B Barber, B Sherrill, M Peeters, J Wiezorek

**Affiliations:** 1 Department of Statistics, RTI Health Solutions, 3040 East Cornwallis Road, Post Office Box 12194, Research Triangle Park, NC 22709-2194, USA; 2 Department of Global Health Economics, Amgen Inc., One Amgen Center Drive, Thousand Oaks, CA 91320-1799, USA; 3 Department of Oncology, University Hospital Antwerp, Wilrijkstraat 10, B-2650 Edegem, Antwerp, Belgium

**Keywords:** panitumumab, metastatic colorectal cancer, quality-adjusted survival

## Abstract

**Background:**

Panitumumab+best supportive care (BSC) significantly improved progression-free survival (PFS) *vs* BSC alone in patients with chemo-refractory wild-type *KRAS* metastatic colorectal cancer (mCRC). We applied the quality-adjusted time without symptoms of disease or toxicity (Q-TWiST) analysis to provide an integrated measure of clinical benefit, with the objective of comparing quality-adjusted survival between the two arms. As the trial design allowed patients on BSC alone to receive panitumumab after disease progression, which confounded overall survival (OS), the focus of this analysis was on PFS.

**Methods:**

For each treatment group, the time spent in the toxicity (grade 3 or 4 adverse events; TOX), time without symptoms of disease or toxicity (TWiST), and relapse (after disease progression; REL) states were estimated by the product-limit method, and adjusted using utility weights derived from patient-reported EuroQoL 5-dimensions measures. Sensitivity analyses were performed in which utility weights (varying from 0 to 1) were applied to time in the TOX and REL health states.

**Results:**

There was a significant difference between groups favouring panitumumab+BSC in quality-adjusted PFS (12.3 weeks *vs* 5.8 weeks, respectively, *P*<0.0001) and quality-adjusted OS (*P*=0.0303).

**Conclusion:**

In patients with chemo-refractory wild-type *KRAS* mCRC, panitumumab+BSC significantly improved quality-adjusted survival compared with BSC alone.

Despite significant improvements in recent years, the treatment of metastatic colorectal cancer (mCRC) for the vast majority of patients is rarely curative, and thus the aims of treatment in this setting extend beyond prolongation of survival to include the improvement of tumour-related symptoms, prevention of tumour progression and/or maintenance of quality of life (Van Cutsem *et al*, 2010). Analyses of clinical trials for mCRC, however, typically focus on efficacy and safety outcomes independently, which can complicate the quantitative evaluation of the overall benefits of treatment. Panitumumab, a fully human monoclonal antibody directed against the epidermal growth factor receptor (EGFR), has been shown to delay disease progression in combination with best supportive care (BSC) compared with BSC alone in patients with chemo-refractory wild-type *KRAS* mCRC (Amado *et al*, 2008). However, similar to other monoclonal antibodies used in cancer therapy, panitumumab is also associated with a well-defined adverse event (AE) profile: in particular, skin toxicity, a well-known side effect of anti-EGFR monoclonal antibodies, is observed in up to 90% of patients (Van Cutsem *et al*, 2007).

The quality-adjusted time without symptoms of disease or toxicity of treatment (Q-TWiST) analysis reflects that cancer therapy is frequently associated with toxicities that may reduce the benefits of increased survival (Miyamoto and Eraker, 1985; Goldhirsch *et al*, 1989; Glasziou *et al*, 1990; Cole *et al*, 1994; Miyamoto, 1999). Quality-adjusted time without symptoms of disease or toxicity incorporates progression, survival, treatment toxicities and utility measures into a single metric providing an integrated measure of clinical benefit. The analysis is usually applied to overall survival (OS) data obtained from clinical trials (Goldhirsch *et al*, 1989). However, in the phase 3 trial of panitumumab in chemo-refractory mCRC, patients in the BSC alone arm were allowed to cross over to receive panitumumab after disease progression. Overall, cross-over to panitumumab occurred in 76% of patients in the BSC alone arm, and this confounded the OS data from the study (Van Cutsem *et al*, 2007, 2010; Amado *et al*, 2008). Progression-free survival (PFS) was the primary end point of the phase 3 trial. In order to better characterise the clinical benefits of panitumumab in mCRC, we, therefore, performed a Q-TWiST analysis to compare quality-adjusted survival among subjects with chemo-refractory wild-type *KRAS* mCRC receiving panitumumab+BSC with that of BSC alone, with a focus on the PFS as OS was confounded by the cross-over study design.

## Materials and methods

### Data source

This Q-TWiST analysis was performed using PFS and OS data from patients with wild-type *KRAS* tumours collected during the phase 3, open-label, randomised, controlled study comparing the use of panitumumab+BSC with BSC alone in patients with chemo-refractory mCRC. The patient population and design for this trial have been described elsewhere (Van Cutsem *et al*, 2007; Amado *et al*, 2008). Briefly, patients with EGFR-detectable mCRC and documented evidence of disease progression after failure of fluoropyrimidines and prespecified exposure to oxaliplatin and irinotecan were randomly assigned to receive panitumumab 6 mg kg^−1^+BSC every 2 weeks or BSC alone until disease progression, inability to tolerate the investigational product or discontinuation for other reasons. Patients in the BSC alone arm could receive panitumumab after disease progression.

The primary end point of the study was PFS, with progression assessed by central radiologic review (not by investigator's evaluation) at specified time points from weeks 8 to 48 using Response Evaluation Criteria in Solid Tumours (RECIST), then every 3 months thereafter. Overall survival was a secondary end point. All subjects continued to be followed for survival approximately every 3 months for up to 2 years after their randomisation into the study. Safety was assessed every 2 weeks and at a 30-day follow-up visit. Health-related quality of life was reported by patients at baseline and monthly until disease progression using the EuroQoL 5-dimensions (EQ-5D) index (Shaw *et al*, 2005; Odom *et al*, 2011). The EQ-5D index provides a utility score.

### Definitions

Overall survival for each treatment group was partitioned into three health states: toxicity (TOX), time without symptoms of disease or toxicity (TWiST) and relapse (disease progression; REL) (Gelber and Goldhirsch, 1986; Goldhirsch *et al*, 1989). Toxicity was defined as the time spent with grade 3 or 4 AEs prior to disease progression. Per convention, AEs occurring after disease progression were not included in the TOX state. The calculation of TOX was based on all grade 3 or 4 AEs reported in the trial. Each AE had a start date and the end date was when the AE was resolved; otherwise, the end date was truncated at disease progression. A day with multiple AEs was only counted once. The duration of TOX included the total number of days spent with AEs from randomisation to disease progression. Time without symptoms of disease or toxicity was defined as the remaining time prior to disease progression in which no grade 3 or 4 AEs were experienced. The end of the TWiST period was the earliest date of disease progression or death and was censored at the date of the last evaluable disease assessment. For subjects who withdrew because of disease progression that was not confirmed by the Independent Review Committee, radiographic data collected during long-term follow-up was used in the primary analysis of PFS. Relapse (disease progression) was defined as the period following disease progression until death or end of follow-up. This means that for patients in the BSC alone group who crossed over to receive panitumumab after disease progression, any time spent with grade 3 or 4 AEs associated with panitumumab treatment was included in the REL period of the BSC alone arm.

To conduct the Q-TWiST analyses, the patient-reported health status directly assessed in the study with the EQ-5D was used to derive patient-reported utility weights, a method that has been previously reported in the published literature (Bernhard *et al*, 2004; Zbrozek *et al*, 2010). For each patient, EQ-5D assessments were averaged during the following periods: when the patient was experiencing an AE (during TOX); prior to disease progression without AEs (during TWiST) and on or after the date of disease progression (during REL). Specifically, if a patient was experiencing a grade 3 or 4 AE during their monthly visit when EQ-5D was assessed, then the EQ-5D score for that visit was used to derive the utility weights for the TOX period. The available EQ-5D scores for the TOX, TWiST and REL states were then averaged for each treatment arm and used as utility scores in the Q-TWiST calculation. As Q-TWiST analyses often use a range of hypothetical utility values to generate quality-adjusted states (Gelber *et al*, 1996; Irish *et al*, 2005; Konski *et al*, 2009), a sensitivity analysis using hypothetical utility values was also performed.

### Statistical analysis

#### Estimation of health state durations

The product-limit method (Kaplan and Meier, 1958) was used to estimate the mean amount of time in the following states: time with any toxicity after randomisation but prior to progression (i.e., TOX); time from randomisation to progression or death (i.e., PFS) and time from randomisation until death from any cause (i.e., OS). Survival curves that corresponded to TOX, PFS and OS were plotted on the same graph for each treatment group. The areas between the curves represented the restricted mean durations of TWiST and REL as follows: 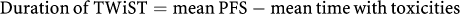




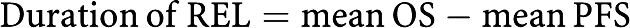



#### Calculation of Q-TWiST

The mean Q-TWiST for each treatment arm was calculated using the following formulae: 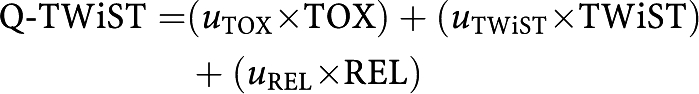 where *u*_TOX_, *u*_TWiST_ and *u*_REL_ represented the average group utility values for each health state and TOX, TWiST and REL represented the mean duration of the health states. The Q-TWiST scores for each treatment group portrayed the quality-adjusted survival experienced by patients during this study. Obviously, TOX and TWiST together represent the PFS and Q-TWiST analysis becomes the quality-adjusted PFS analysis in the case when the utility for the period of REL (i.e., *u*_REl_) is set to zero. Differences between treatment groups (panitumumab+BSC *vs* BSC alone) in mean Q-TWiST were calculated. A 95% confidence interval (CI) and two-sided *P*-value for testing the null hypothesis of no difference between treatment groups was performed based on the normal approximation (*Z*-test) with standard errors calculated by the bootstrap method. The bootstrap was conducted by repeated sampling, with replacement, from the sample of patients included in the study, to obtain a new sample. The means for the new sample were calculated from the area under the Kaplan–Meier curve. This process was repeated 1000 times. Based on the means obtained by the bootstrap, the standard errors were calculated (Glasziou *et al*, 1990).

#### Sensitivity analysis

Sensitivity analyses were performed based on the threshold utility analyses, in which utility weights (varying from 0 to 1) were applied to time in the TOX and REL health states, while holding the utility of TWiST at 1 (representing the highest utility that can be expected for a patient with mCRC) (Goldhirsch *et al*, 1989; Gelber *et al*, 1995b; Cole *et al*, 1996). All analyses were performed using SAS statistical software Version 9.1.3 (SAS, Cary, NC, USA).

## Results

The intention to treat population included 463 patients (Van Cutsem *et al*, 2007). *KRAS* status was ascertained in 427 (92%) of patients. Of these, 243 patients had wild-type *KRAS* tumours (panitumumab+BSC, *n*=124; BSC alone, *n*=119) and were included in the Q-TWiST analyses. Baseline demographics of these patients have been described elsewhere and were well balanced between groups: median age was 63 years and 69% of patients had colon rather than rectal cancer in both groups (Amado *et al*, 2008). A total of 54% (67 out of 124) of panitumumab+BSC patients and 27% (32 out of 119) of BSC alone patients experienced a ⩾grade 3 AE prior to progression or censoring for progression. Of the 119 BSC alone patients, 90 (76%) went on to receive panitumumab after disease progression and 20 had a complete or partial response during panitumumab treatment (Amado *et al*, 2008).

### Patient-reported utility data

Observed utility data for each health state based on the EQ-5D index are shown in Table 1. Ninety-three per cent (225 out of 243) of patients completed at least one EQ-5D assessment. The average utility value observed during the TOX state (⩾grade 3 AE) was 0.60 for the panitumumab+BSC group and 0.44 for the BSC alone group. The average utility for the TWiST state was higher for the panitumumab+BSC group compared with BSC alone (0.76 *vs* 0.66, respectively), while utility values were similar in the two groups for the REL state.

### Health state durations

**Figure 1:**
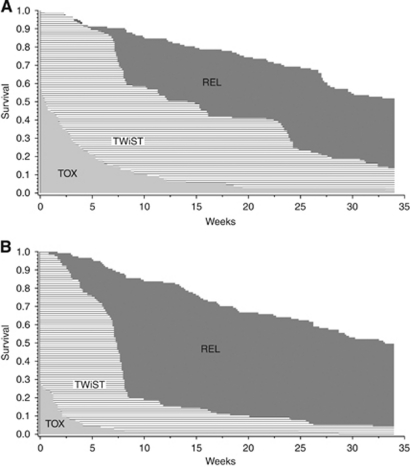
Partitioned survival curves for (**A**) panitumumab+BSC and (**B**) BSC alone. BSC=best supportive care; REL=relapse period until death or end of follow up; TOX=days with ⩾grade 3 adverse events; TWiST=time without symptoms or toxicity.

Partitioned survival plots for panitumumab+BSC and BSC alone, restricted to median follow-up, are presented in Figure 1, with the estimated mean duration of each health state shown in Table 2. The mean duration in the TOX state was approximately 3.5 weeks in the panitumumab+BSC group compared with 1.1 weeks for the BSC alone treatment group (*P*=0.0006); however, patients on panitumumab+BSC spent 13.3 weeks in the TWiST state compared with 8.0 weeks for patients on BSC alone and the difference of 5.3 weeks was statistically significant (*P*<0.0001). Patients on BSC alone had a longer duration during REL; however, the duration of REL for BSC alone was confounded by the cross-over design of the trial.

### Q-TWiST analysis

**Figure 2:**
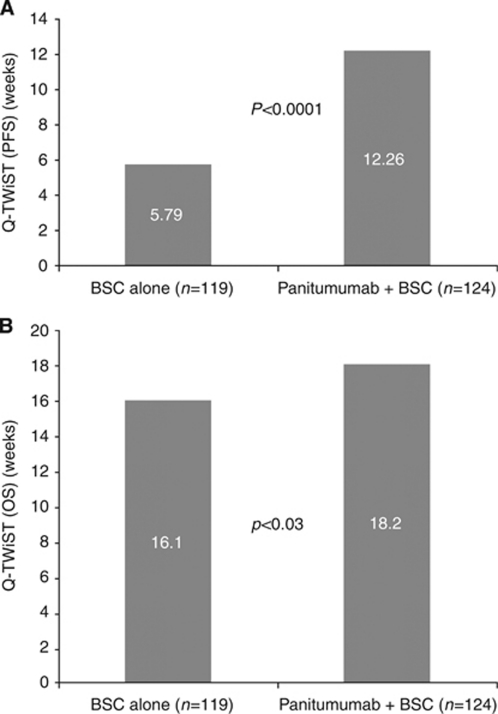
(**A**) Mean quality-adjusted PFS for patients with wild-type *KRAS* mCRC receiving panitumumab+BSC or BSC alone. (**B**) Mean quality-adjusted OS for patients with wild-type *KRAS* mCRC receiving panitumumab+BSC or BSC alone. BSC=best supportive care; mCRC=metastatic colorectal cancer; OS=overall survival; PFS=progression-free survival; Q-TWiST=quality-adjusted time without symptoms of disease or toxicity of treatment.

Applying the utility values from Table 1 to the duration of the TOX and TWiST states, the quality-adjusted difference between groups in PFS was 6.5 weeks favouring panitumumab+BSC over BSC alone (12.3 *vs* 5.8 weeks, respectively), which was statistically significant (*P*<0.0001) (Figure 2). Following incorporation of the REL state into the calculation, overall Q-TWiST was 18.2 weeks for panitumumab+BSC compared with 16.1 weeks for BSC alone. Despite the fact that the duration of the REL state was confounded by the significant degree of cross-over to panitumumab after disease progression in those patients randomised to BSC alone arm, the difference in Q-TWiST between groups (panitumumab+BSC *vs* BSC alone) was statistically significant (*P*=0.0303) with a point estimate of 2.1 weeks.

### Sensitivity analysis

Using a range of hypothetical utility weights for the TOX and setting the utility for the REL to zero (i.e., the first five rows in Table 3), differences in quality-adjusted PFS between the two treatment groups ranged from 5.3 to 7.6 weeks, favouring panitumumab+BSC. All these differences were also statistically significant (*P*<0.0001).

**Figure 3:**
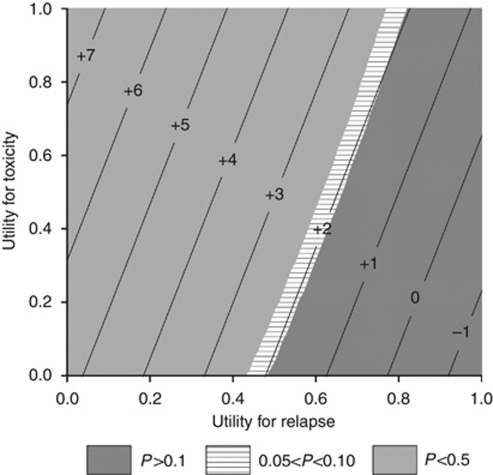
Threshold utility plot showing differences in Q-TWiST (in weeks) at varying utility levels. *Note*: Positive numbers indicate a longer duration of Q-TWiST for patients in combination therapy (panitumumab+BSC).

Using varying hypothetical utility weights for both TOX and REL health states, the overall Q-TWiST difference between groups ranged from −1.6 to 7.6 weeks and favoured panitumumab+BSC therapy for 22 of 25 (or 88% of possible value combinations) hypothetical utility levels (Table 3; Figure 3). Results were statistically significant for all TOX utility levels when REL was valued at ⩽0.5 except utility of TOX=0 and REL=0.5.

## Discussion

The major goals of treatment for mCRC are prolongation of survival, improvement of tumour-related symptoms, prevention of tumour progression and/or maintenance of quality of life (Van Cutsem *et al*, 2010). In chemo-refractory wild-type *KRAS* mCRC, panitumumab+BSC provided statistically significant improvements in PFS compared with BSC alone (hazard ratio, 0.45; 95% CI: 0.34–0.59) (Amado *et al*, 2008). However, as dermatological toxicity is a well-known side effect of anti-EGFR monoclonal antibodies, including panitumumab (Li and Perez-Soler, 2009), we considered that it would be particularly valuable to apply the Q-TWiST analysis to these results, thus combining efficacy and safety measures, and allowing a direct evaluation of the impact of treatment toxicities on patient experience (Goldhirsch *et al*, 1989; Glasziou *et al*, 1990; Gelber *et al*, 1995a; Cole *et al*, 2004).

In our analysis, using utility scores based on EQ-5D assessments collected during the phase 3 study, patients with wild-type *KRAS* tumours receiving panitumumab+BSC compared with patients on BSC alone had 6.5 more quality-adjusted weeks for PFS. These results closely reflect the difference in unadjusted PFS reported by Amado *et al* (2008), but extend our understanding to suggest that toxicities associated with panitumumab, such as dermatological events, are more than offset by the significantly extended time in the TWiST state compared with BSC alone. We focused on the period prior to progression, that is PFS, because of the inherent limitations of the phase 3 study design, which allowed patients randomised to BSC alone arm to cross over to panitumumab after disease progression (Van Cutsem *et al*, 2007). However, despite these limitations, a difference in overall quality-adjusted survival between arms was identified. Although relatively small, this difference was also statistically significant in favour of panitumumab+BSC. The evaluation of a clinically important difference for Q-TWiST has been studied (Revicki *et al*, 2006). Based on an analysis of Q-TWiST studies in oncology, it was recommended that a Q-TWiST difference of 10% or more be considered clinically important, with differences of 15% or more being clearly clinically important. In the current study, the differences in quality-adjusted PFS between the two treatment groups ranged from 5.3 to 7.6 weeks longer for panitumumab+BSC compared with BSC alone, representing a 73–104% increase in the median PFS of 7.3 weeks seen in the BSC alone arm (Amado *et al*, 2008). These values would be considered clearly clinically important according to Revicki *et al* (2006). Despite the significance of the relative difference, however, the absolute improvement was rather modest.

In the primary analysis, we used utility scores based on actual EQ-5D assessments made during this study. We noted that EQ-5D scores were higher for patients on panitumumab+BSC compared with patients on BSC alone during periods of both TOX and TWiST, which is supported by the demonstration of better maintenance of overall HRQoL in patients on panitumumab+BSC compared with BSC alone (Odom *et al*, 2011). The most frequent toxicity of panitumumab is skin rash (Van Cutsem *et al*, 2007). Despite the perceived detrimental impact of skin rash on HRQoL (Lynch *et al*, 2007), the higher EQ-5D during periods of TOX in patients on panitumumab+BSC is consistent with the reported association between more severe skin toxicity and higher rather than lower HRQoL scores (Peeters *et al*, 2009). More severe skin toxicity following panitumumab treatment has been also associated with improved outcomes (Peeters *et al*, 2009; Douillard *et al*, 2010).

Basing quality adjustments on patients’ experience of the treatments administered during the clinical trial is a strength of this analysis in that it does reflect the actual utilities obtained. There are also associated limitations, as EQ-5D scores were not reported for all patients or all visits. However, we performed a sensitivity analysis to assess anticipated Q-TWiST differences across of range of possible utilities – an approach that is often used as the core method in Q-TWiST analyses reported in the literature (Gelber *et al*, 1996; Irish *et al*, 2005; Konski *et al*, 2009). This analysis provides an assessment of how patient preferences for trade-offs in disease control compared with tolerability concerns might influence treatment choice. Varying the utility weight for the TOX state between 0 and 1 consistently showed that the quality-adjusted PFS for panitumumab+BSC was significantly longer than that of the BSC alone arm. Similar sensitivity analyses were also performed for overall quality-adjusted survival. Since the utility for the REL state determined from the clinical trial data was based on EQ-5D values at withdrawal (or was censored if these data were unavailable), these values may not reflect realistic values for the entire period after disease progression during which significant deterioration in health is anticipated. In the threshold utility analysis, panitumumab+BSC therapy was favoured over BSC alone for most of the hypothetical utility levels applied to the Q-TWiST calculations. As shown in Table 3, the most pronounced differences favouring panitumumab treatment were seen when the REL state was valued at low levels. However, the gain for using panitumumab decreased as the utility weights for REL increased (>0.5), especially as the utility weights of time in TOX also decreased.

It is worthwhile to note that this Q-TWiST analysis was based on primary PFS data that demonstrated a significant improvement in PFS for panitumumab+BSC over BSC alone. A greater improvement in PFS was seen for panitumumab+BSC *vs* BSC alone based on the PFS analysis by the Committee for Medicinal Products for Human Use, in which unscheduled tumour assessments were moved to the nearest scheduled time point. Thus, if a Q-TWiST analysis was based on the latter PFS, we would likely expect a larger advantage in quality-adjusted PFS. Also, since the period of REL, and thus the OS, was confounded by the cross-over design of the trial, it was likely the quality-adjusted OS benefit of panitumumab derived from this Q-TWiST analysis might underestimate the true OS benefit of panitumumab.

Quality-adjusted time without symptoms of disease or toxicity of treatment can be considered as an alternative to the quality-adjusted life years (QALY), which is the most established method of combining quality and quantity of life used extensively in cost-effectiveness analysis (Miyamoto and Eraker, 1985; Miyamoto, 1999). In Q-TWiST analysis, utility scores are assigned to a number of discrete pre-defined ‘states’ (such as TOX, REL) that each patient may experience, which allows flexibility on a study-by-study basis. However, of course, this generates inconsistencies between studies and so, unlike QALYs, it is impossible to compare across studies or disease groups unless exactly the same scheme is used. There are also frequently concerns about the selection of Q-TWiST states and utilities, particularly for the latter, which may be very arbitrary and often questionable. Nevertheless, the Q-TWIST concept is an innovative method of adjusting survival to account for different patient experiences, and can be based on clinical information, and calculated without prospective questionnaires. To mitigate concerns regarding the selection of utilities, sensitivity analyses were conducted based on the threshold utility analyses as described in the literature (Goldhirsch *et al*, 1989; Gelber *et al*, 1995b; Cole *et al*, 1996). Some additional limitations of the study also need to be acknowledged. First, the study followed the standard approach of the Q-TWiST analysis, in which only grade 3 or 4 AEs were taken into considered in the calculation of the TOX period. However, a sensitivity analysis that incorporated grade 2 AEs was conducted and consistent findings were observed (data not shown). Second, for the TOX period, a day with multiple AEs was only counted once and AEs were not differentiated based on their types. Third, in the primary analysis, patient-reported EQ-5D data were used to derive utility weights. Ideally, EQ-5D should have been assessed daily, so the utility weights could be derived from all days when AEs were being experienced. In fact, EQ-5D was not reported by all patients at all visits (Odom *et al*, 2011) and was assessed only monthly. Despite these limitations, the estimated utility values for different states (TOX, TWiST or REL) were in the range as reported in the literature (Earle *et al*, 2000; Sherrill *et al*, 2008), and the difference between panitumumab+BSC *vs* BSC alone was also consistent with the overall better HRQoL as measured by EQ-5D reported by Odom *et al* (2011).

In conclusion, in patients with wild-type *KRAS* mCRC, panitumumab+BSC provided significant improvements in quality-adjusted PFS and quality-adjusted OS compared with BSC alone, illustrating that the toxicities associated with panitumumab are more than offset by the associated increase in time without severe toxicity.

## Figures and Tables

**Table 1:** Average utility values by health state for patients with wild-type *KRAS* tumours receiving panitumumab+BSC or BSC alone, based on the EQ-5D index

	**Panitumumab+BSC (*n*=124)**		**BSC alone (*n*=119)**	
**Health state**	***n***	**Utility**	***n***	**Utility**
TOX	37	0.6008	13	0.4409
TWiST	104	0.7678	103	0.6630
REL	68	0.6318	63	0.6407

Abbreviations: BSC=best supportive care; EQ-5D=EuroQol-5 dimensions; REL=relapse period until death or end of follow-up; TOX=days with ⩾grade 3 adverse events; TWiST=time without symptoms or toxicity.

**Table 2:** Mean duration of health states for patients with wild-type *KRAS* tumours receiving panitumumab+BSC or BSC alone

	**Mean duration (weeks)**			
**Health state**	**Panitumumab+BSC (*n*=124)**	**BSC alone (*n*=119)**	**Difference (Panit+BSC) *vs* BSC**	***P*>∣*Z*∣** a
TOX	3.47	1.09	2.37	0.0006
TWiST	13.26	8.01	5.25	<0.0001
REL	9.35	16.15	−6.80	<0.0001

Abbreviations: BSC=best supportive care; Panit=panitumumab; REL=relapse period until death or end of follow-up; TOX=days with ⩾grade 3 adverse events; TWiST=time without symptoms or toxicity.

a Null hypothesis: difference (panitumumab+BSC – BSC alone)=0.

**Table 3:** Duration and significance of differences between the panitumumab+BSC and BSC alone groups in Q-TWiST at varying utility weights

**Utility per phase**			**Panitumumab+BSC *vs* BSC alone**		
**TOX**	**TWiST**	**REL**	**Difference in Q-TWiST (weeks)**	***P-*value**	**95% CI**
0.00	1.00	0.00	5.25	<0.0001	2.90, 7.59
0.25	1.00	0.00	5.84	<0.0001	3.55, 8.13
0.50	1.00	0.00	6.43	<0.0001	4.15, 8.72
0.75	1.00	0.00	7.03	<0.0001	4.70, 9.36
1.00	1.00	0.00	7.62	<0.0001	5.20, 10.04
0.00	1.00	0.25	3.55	0.0020	1.30, 5.80
0.25	1.00	0.25	4.14	0.0002	1.97, 6.31
0.50	1.00	0.25	4.73	<0.0001	2.60, 6.87
0.75	1.00	0.25	5.33	<0.0001	3.17, 7.48
1.00	1.00	0.25	5.92	<0.0001	3.69, 8.15
0.00	1.00	0.50	1.84	0.1205	−0.48, 4.17
0.25	1.00	0.50	2.44	0.0316	0.21, 4.66
0.50	1.00	0.50	3.03	0.0061	0.86, 5.20
0.75	1.00	0.50	3.63	0.0010	1.46, 5.79
1.00	1.00	0.50	4.22	0.0002	2.01, 6.43
0.00	1.00	0.75	0.14	0.9124	−2.42, 2.71
0.25	1.00	0.75	0.74	0.5548	−1.71, 3.19
0.50	1.00	0.75	1.33	0.2716	−1.04, 3.70
0.75	1.00	0.75	1.92	0.1076	−0.42, 4.27
1.00	1.00	0.75	2.52	0.0369	0.15, 4.88
0.00	1.00	1.00	−1.56	0.2964	−4.48, 1.37
0.25	1.00	1.00	−0.96	0.5001	−3.76, 1.84
0.50	1.00	1.00	−0.37	0.7895	−3.08, 2.34
0.75	1.00	1.00	0.22	0.8693	−2.44, 2.89
1.00	1.00	1.00	0.82	0.5476	−1.85, 3.48

Abbreviations: BSC=best supportive care; Q-TWiST=quality-adjusted time without symptoms of disease or toxicity of treatment; REL=relapse period until death or end of follow-up; TOX=days with ⩾grade 3 adverse events; TWiST=time without symptoms or toxicity.
